# Consent and medical treatment: The legal paradigm in India

**DOI:** 10.4103/0970-1591.56202

**Published:** 2009

**Authors:** Omprakash V. Nandimath

**Affiliations:** National Law School of India University, Bangalore 560 072, India

**Keywords:** Autonomy, prior informed consent, valid consent

## Abstract

The element of consent is one of the critical issues in medical treatment. The patient has a legal right to autonomy and self determination enshrined within Article 21 of the Indian Constitution. He can refuse treatment except in an emergency situation where the doctor need not get consent for treatment. The consent obtained should be legally valid. A doctor who treats without valid consent will be liable under the tort and criminal laws. The law presumes the doctor to be in a dominating position, hence the consent should be obtained after providing all the necessary information.

## INTRODUCTION

The element of consent is one of the critical issues in the area of medical treatment today. It is well known that the patient must give valid consent to medical treatment; and it is his prerogative to refuse treatment even if the said treatment will save his or her life. No doubt this raises many ethical debates and falls at the heart of medical law today. The earliest expression of this fundamental principle, based on autonomy, is found in the Nuremberg Code of 1947. The Nuremberg Code was adopted immediately after World War II in response to medical and experimental atrocities committed by the German Nazi regime.[1] The code makes it mandatory to obtain voluntary and informed consent of human subjects. Similarly, the Declaration of Helsinki adopted by the World Medical Association in 1964 emphasizes the importance of obtaining freely given informed consent for medical research by adequately informing the subjects of the aims, methods, anticipated benefits, potential hazards, and discomforts that the study may entail.[2] Several international conventions and declarations have similarly ratified the importance of obtaining consent from patients before testing and treatment. The present paper examines the entire gamut of issues pertaining to consent from the point of view of the legal environment as it exists in India today. The circle of legal development in the area (i.e., consent) appears to be almost complete when the apex court in India recently ruled that, it is not just the ‘consent’ or ‘informed consent’ (as it is known worldwide) but it shall also be ‘prior informed consent’ generally barring some specific cases of emergency. This places a medical professional in a tremendous dilemma. Hence, it is time to revisit the area of ‘consent and medical treatment’ to understand the sensitive and underpinning elements.

## THE LEGAL BASIS OF CONSENT

Consent is perhaps the only principle that runs through all aspects of health care provisions today. It also represents the legal and ethical expression of the basic right to have one's autonomy and self-determination. If a medical practitioner attempts to treat a person without valid consent, then he will be liable under both tort and criminal law. Tort is a civil wrong for which the aggrieved party may seek compensation from the wrong doer. The consequences would be payment of compensation (in civil) and imprisonment (in criminal). To commence, the patient may sue the medical practitioner in tort for trespass to person. Alternatively, the health professional may be sued for negligence. In certain extreme cases, there is a theoretical possibility of criminal prosecution for assault or battery. The traditional definition of battery is an act that directly and either intentionally or negligently causes some physical contact with another person without that person's consent. If a person has consented to contact expressedly or by implication, then there is no battery. It is a rare case in which a doctor would be held liable for criminal breach, unless there is gross disrespect to the patient's bodily autonomy, for instance, if a patient's organs are taken without his consent.

In tort law, usage of force against any human body, without proper justification, is actionable irrespective of the quantum of force. If the medical practitioner attempts to treat a patient without obtaining proper consent, he will be held guilty under tort law. Consent for treatment may be expressed or implied. The patient entering the consultation chambers by his own volition may be considered to have given consent for a clinical diagnosis to be carried out. Consent may be inferred from the general submission by a patient to orders given by a doctor during clinical diagnosis. This is an excellent example of implied consent. During the clinical examination, there might arise the need for an intimate examination of the patient, such as a vaginal examination. For such an examination, the medical practitioner must ideally obtain another consent by asking the patient's permission orally. Furthermore, if there is a need to undergo an invasive examination, such as an incision or drawing of samples of body fluids, a written consent of the patient is ideally required.

Often medical practitioners ask for precise prescriptions for the situations when written consent is needed. It is interesting to note that what law demands is mere consent and not written consent and does not prescribe such requirement on a mandatory basis. In fact, the medical practice itself determines the need for written consent. Ideally, where the patient is subjected to anesthesia (either local or general) or where the patient is subjected to severe pain during administration of the treatment, a written consent would be helpful. There is no mandate that a doctor should always obtain written consent and failure of which would hold him liable. However, if there is written consent, the medical practitioner would have greater ease in proving consent in case of litigation. To standardize the practice, the Medical Council of India (MCI) has laid down guidelines that are issued as regulations in which consent is required to be taken in writing before performing an operation.[3] The MCI guidelines are applicable to operations and do not cover other treatments. For other treatments, the following may be noted as general guidelines:

1. For routine types of treatment, implied consent would suffice
2. For detailed types of treatment, ideally express oral consent may be needed
3. For complex types of treatment, written express consent is required

## CAPACITY AND INFORMATION WHILE SEEKING CONSENT

There are two more additional aspects to be borne in mind: first, valid consent can be obtained only from a patient who is competent to consent and secondly, such consent must also be informed consent. To be competent to give a legally effective consent, the patient must be endowed with the ability to weigh the risks and benefits of the treatment that is being proposed to him. The law presumes that such an ability is generally acquired with the attainment of the age of maturity. A person who has attained the competent age and who has sound mind can give valid consent to the medical practitioner for any treatment. Persons who have attained the age of 18 are generally considered to have attained the age of maturity and are competent to give consent. The law thus presumes capacity, rationality, autonomy, and freedom if the person has attained the age of so called maturity. On the other hand, where there is reason to believe that a patient is unable to understand the nature of the treatment and its benefits or side effects before making the decision, it is necessary to consider whether an adult presumption of capacity is rebutted in that particular case. If the patient is incompetent to give consent, then the consent may be obtained from the attendant of the patient.[4] In the UK, there are several ethical issues raised regarding the proxy consent on behalf of such persons. Even the Law Commission Report (Mental Incapacity, 1995) suggests few reforms. Irrespective of the age, for a person who is incompetent due to unsoundness of mind, consent will be obtained from the guardian of the patient. In India, the court has not come across borderline cases of an adult refusing treatment leading to emergency and leaving the doctor in a dilemma, unlike in the west.[5]

The law also presumes that the medical practitioner is in a dominating position vis-à-vis the patient; hence, it is his duty to obtain proper consent by providing all the necessary information. Consent without necessary information is no consent at all. Unfortunately, the expression ‘informed consent’ is often used without precision. The “informed consent” doctrine is American in origin and relates to the amount of information that a patient should be provided with to avoid any probable action in negligence. Rarely, a medical practitioner or a hospital administrator can rely upon the consent form signed by the patient, when the contention is that he was made to sign on the dotted lines of such format without proving necessary information. This practice is also developed by the practice of treating the consent form as a one of standard forms of contracts and eliminating all such unfair and sweeping clauses, which will only benefit the medical practitioner. It is rather necessary as the pro-forma is prepared by the medical practitioner/hospital administration, and the patient is left with the choice of either accepting it as whole or rejecting it. Therefore, it is absolute imperative that a medical practitioner provide all relevant information relating to the proposed treatment to the patient in a language understandable to him, while obtaining the much needed consent for the treatment.

However, the nature of the information that a patient must have in order to give informed consent is a debatable question, as the American and English viewpoints differ to some extent. Informed consent from the American sense is often described from the viewpoint of a prudent patient, popularly know as the prudent patient test. In this approach, the highest respect for the patient's right of self-determination about a particular therapy is recognized. This will lead to a so-called objective test of disclosure wherein the doctor will keep in mind the patient and disclose all such information which is required to be given. In other words, there is a presumption that some standard information is required to be disclosed to every patient, and the extent of such disclosure is neither left to the discretion of the doctor (of course leaving out special circumstances where the doctor might have strong reasons for concealing) nor he can rely upon the defense of disclosure like a reasonable medical practice or practitioner.[5] In contrast to this, the English approach is doctor centric, which is also popularly narrated as the prudent doctor test of disclosure. Here, the doctor is taken as a professional-man endowed with greater prudence to protect the right interest of the patient and bestowed with the final right to decide what information shall be divulged to the patient considering the circumstances and how much information is to be divulged. Lord Templeman in ‘Sidway’ encapsulated this as follows:

“When the doctor himself is considering the possibility of a major operation, the doctor is able with his medical training, with his knowledge of the patient's medical history, and with his objective position to make a balanced judgment as to whether the operation should be performed or not. The duty of the doctor in these circumstances, subject to his overriding duty to have regard to the best interests of the patient, is to provide the patient with information which will enable the patient to make a balanced judgment if the patient chooses to make a balanced judgment”.[6]

Finally, whatever might be the difference of approach it is evident that a medical practitioner is obligated to provide the necessary information before obtaining consent from a patient. To account for the Indian position, although we do not have much litigation, unlike in the West, it may be concluded that the courts have assigned immense significance to the requirement of informed consent. A medical practitioner in India has a duty to provide all the necessary information to the patient in a language that is understandable to him. Regarding the quantum of information, there are no clear parameters laid down by the courts. Therefore, it is reasonable information which a doctor deems fit considering best practices. Considering the knowledge gap in this regard, the professional regulatory body for medicine can play an important role in establishing standards.

## INDIAN LAW ON CONSENT

The principle of autonomy is enshrined within Art. 21 of the Indian Constitution, which deals with the right to life and personal liberty. The expression personal liberty under Art. 21 is of the widest amplitude and covers a wide variety of rights, including the right to live with human dignity and all that goes along with it, and any act which damages, injures, or interferes with the use of any limb or faculty of a person, either permanently or temporarily.[7] However, the common law application of consent is not fully developed in India, although the Indian courts have often referred to these principles. In such situations, obviously one has to refer to the principles of the Indian Contract Act and the Indian Penal Code. The relationship between a medical professional and his patient is a contract by parties competent to contract giving rise to contractual obligations. Parties are generally competent (in accordance with the Indian Majority Act) (i) if they have attained the age of 18, (ii) are of sound mind, and (iii) are not disqualified by any law to which they are subject to. Furthermore, there is a stipulation in the contract law stating that consent of any party (in our case it is the patient) that is obtained by coercion, undue-influence, mistake, misrepresentation or fraud, will render the agreement invalid. However, in England, the General Medical Council guidelines state that the consenting age is 16 years old. A young person can be treated as an adult and can be presumed to have the capacity to decide. If the child is under the age of 16 he or she may have the capacity to decide, depending on his/her ability to understand what is involved. Where a competent child refuses treatment, a person with parental responsibility or the court may authorize investigation or treatment which is in the child's best interests. Interestingly, the position is different in Scotland where those with parental responsibility cannot authorize procedures a competent child has refused.

The consent obtained, of course, after getting the relevant information will have its own parameter of operation to render protection to the medical practitioner. If the doctor goes beyond these parameters, he would be treating the patient at his risk, as it is deemed that there is no consent for such treatment at all. A doctor who went ahead in treating a patient, to protect the patient's own interest, was held liable as he was operating without consent.[8] The patient was suspected to have appendicitis. After obtaining due consent, she was subjected to an operation. However, upon incision, it was found that her appendix was normal and not inflamed. To protect the interest of the patient, the doctor removed her gangrenous gall bladder. Later, it was discovered that the kidney of the patient was affected. The doctor was held liable as he was operating without consent. This case law also signifies the traditional notion of paternalism prevalent among the members of the medical fraternity. It is a notion where the doctor takes-up the role of a parent of the patient and starts deciding on behalf of the patient himself. Unfortunately, the law does not accept this notion. The first priority of law is always the right of autonomy of the patient provided he is endowed with necessary capacity. A medical practitioner who believes that a medical procedure is appropriate and necessary for a patient's well being can perhaps be forgiven for believing that the principle of autonomy should be sacrificed in the best interest of the patient. In the present case, had the doctor stopped after realizing that the patient's appendix was normal, he would have been protected as he was working under the valid consent of the patient, and more importantly, mere error of judgment is not culpable. When he proceeded in removing her gall bladder, he was acting *sans* valid consent, which was an extreme case of professional paternalism and gross disobedience to the right of the patient's autonomy. Hence, some commentators like Mill, *et al.* have advocated for minimal level of paternalism in the interest of the medical profession and the overall inability of humans in taking rational decisions, during the time of crises.[9]

Regarding proxy consent, when the patient is unable to give consent himself, there are no clear regulations or principles developed in India. If such a situation exists, the medical practitioner may proceed with treatment by taking the consent of any relative of the patient or even an attendant. In one case, the wife of a patient informed the hospital authorities in unambiguous terms that she had no objection to her husband undergoing bypass surgery, her consent was deemed sufficient for the purpose of any formalities with which the hospital was required to comply.[10]

Interestingly, in another case the relationship between the patient and his wife were strained. A patient was operated on for sterilization. While giving consent he deposed that he is married and has two baby girls. In fact, he was undergoing an operation only for getting the money as incentive. After the operation, his father contended that the patient was of unstable mind and was not competent to give consent. The court held that if there are no circumstances for a doctor to sense foul play or doubtabout the capacity of the patient, he is protected.[11] These two cases demonstrate that a doctor acting reasonably under normal circumstances is always protected and he is never expected to play the role of an investigative agency.

Recently, the apex court gave an impacting judgment in the area. Wherein the court observed that “where a surgeon is consulted by a patient and consent of the patient is taken for diagnostic procedure/surgery, such consent can't be considered as authorization or permission to perform therapeutic surgery either conservative or radical (except in a life-threatening emergent situation)”.[12] For the fist time in India, the court ruled that however broad consent might be for diagnostic procedure, it can not be used for therapeutic surgery. Furthermore, the court observed that “where the consent by the patient is for a particular operative surgery it can't be treated as consent for an unauthorized additional procedure involving removal of an organ only on the ground that it is beneficial to the patient or is likely to prevent some danger developing in the future, where there is no imminent danger to the life or health of the patient”. This proposition puts fetter upon the role of a “paternal doctor” in the Indian scenario. In one case, a 44-year-old unmarried female consulted her doctor and was advised to undergo a laparoscopy. A few consent forms were taken from her of which one was for admission and another one was for the surgery. The relevant one among such consent forms gave the doctor an allowance to carry out a “diagnostic and operative laparoscopy” and there was an additional endorsement that a “laparotomy may be needed”. When the patient was in the operation theater (and was unconscious), another proxy consent was taken from her attending mother for a hysterectomy. Her uterus, ovaries, and fallopian tubes were removed. Subsequently, when an action was brought, it was held that the operation was conducted without real consent and the doctors were held liable.

This decision is of very far reaching consequences, pushing the development of consent law to new heights. It is contended that it is not only informed consent which is imperative now, but the same shall be “prior informed consent” unless there is imminent threat to the patient's life. In addition, this decision curtails the scope of proxy consent from the person having parental authority or an attendant.

## EMERGENCY SITUATION AND CONSENT

Interestingly, in India, the entire gamut of laws on consent turns into complex propositions if an emergency medical situation arises. In a few of the milestone decisions, the apex court ruled that a medical practitioner has a duty to treat a patient in an emergency. Emphasizing the paramount duty of any “welfare state“, the Supreme Court stated that Art. 21 imposes an obligation on the State to safeguard the right to life of every person. Preservation of human life is thus of paramount importance. The government hospitals run by the state are bound by duty to extend medical assistance for preserving human life. Failure on the part of a government hospital to provide timely medical treatment to a person in need of such treatment results in the violation of his right to life guaranteed under Art. 21.[13] Proceeding in the same direction, the court emphasized further that every doctor whether at a Government hospital or otherwise has the professional obligation to extend his services with due expertise for protecting life. No law or state action can intervene to avoid or delay the discharge of the paramount obligation cast upon members of the medical profession. The obligation of a doctor is total, absolute, and paramount. Laws of procedure whether in statutes or otherwise that would interfere with the discharge of this obligation cannot be sustained and must, therefore, give way.[14] In one case, the apex court laid down some important guidelines such as (i) The doctor when approached by an injured person, shall render all such help which is possible for him at that time, including referring him to the proper experts, (ii) the doctor treating such persons shall be protected by law, as they are not contravening any procedural laws of the land (regarding jurisdictions etc.), and (iii) all legal bars (either real or perceived by the doctors) are deemed to have been eliminated by the verdict. This is in consonance with the hypocratic oath, which a doctor takes when entering the profession. Hence, a doctor is duty-bound to treat a patient in the case of an emergency, without waiting for any formalities. There are several statutes (like medical institutions regulation acts in various states) imposing this duty upon medical establishments to treat emergency patients, especially accident victims.

The initial proposition (and the attempt of the Supreme Court) is quiet understandable as the doctor has to do his best to save life in emergency situations. This is irrespective of complying with any of the formalities, including consent. Hypothetically, if a patient in an emergency resists taking treatment, what shall be the way out? Indian courts are not very clear on that. The above decisions are delivered keeping in mind the accident victims who were denied medical treatment by doctors, terming them as medico-legal cases. Moreover, in the above instances, the patient would go himself, or be taken by someone (due to an unconscious state) to the doctor to seek medical treatment.

In *Dr. T.T. Thomas vs. Elisa*,^(15)^ the patient was admitted into the hospital on March 11, 1974. Upon admission, the patient was diagnosed as a case of perforated appendix with peritonitis requiring an operation. But, unfortunately no operation was done until his death on March 13, 1974. The contention of the doctor was that no surgery could be adhered to, albeit the suggestion, because the patient did not consent for the surgery. Therefore, other measures were taken to ameliorate the condition of the patient, which grew worse by the next day. Although the patient was then willing to undergo the operation, his condition did not permit it. On the other hand, the version of the respondent (i.e., the Plaintiff) was that the doctor demanded money for performing the surgery. Furthermore, the doctor was attending to some chores in an outside private nursing home to conduct operations on the other patients and that the appellant doctor came back only after the death of the patient. The two versions before the court were: 1) the plaintiff (the deceased patient's wife) said that the doctors concerned demanded a bribe, hence the operation was delayed until it proved fatal and 2) the version of denial for consent. Finally, the court delivered a verdict in favor of the plaintiffs stating that consent under such an emergent situation is not mandatory.[15] It is interesting to note the following observations:

“The consent factor may be important very often in cases of selective operations, which may not be imminently necessary to save the patient's life. But there can be instances where a surgeon is not expected to say that ‘I did not operate on him because, I did not get his consent’. Such cases very often include emergency operations where a doctor cannot wait for the consent of his patient or where the patient is not in a fit state of mind to give or not to give a conscious answer regarding consent. Even if he is in a fit condition to give a voluntary answer, the surgeon has a duty to inform him of the dangers ahead of the risks involved by going without an operation at the earliest time possible”.

“When a surgeon or medical man advances a plea that the patient did not give his consent for the surgery or the course of treatment advised by him, the burden is on him to prove that the non-performance of the surgery or the non-administration of the treatment was on account of the refusal of the patient to give consent thereto. This is especially so in a case where the patient is not alive to give evidence. Consent is implicit in the case of a patient who submits to the doctor and the absence of consent must be made out by the patient alleging it”.

Finally, as stated above, before holding the doctor liable, the court said that “we also hold that the failure to perform an emergency operation on the deceased on 11-3-1974 amounts to negligence and the death of the deceased was on account of that failure”. This decision makes the entire discussion of consent law more complex. Although this case law can't be given more accent (because it is a High Court decision), the viewpoint is an interesting one to note. In light of all these developments, it may be concluded that there are many grey areas in this field of consent law in India, which can be eliminated by pro-active intervention by the concerned professional regulatory body.
